# The Fitzgerald Colony*The above letter we reproduce, with the permission of the writer, Dr. Coe, from the *Northwestern Lancet*, of St. Paul. It is a full refutation of some of the reports in circulation about the colony. Dr. Coe writes us that “this article was true when I wrote it, and it is true now.”

**Published:** 1896-04

**Authors:** S. B. Coe

**Affiliations:** Fitzgerald, Ga.


### CORRESPONDENCE.

### THE FITZGERALD COLONY.*

FITZGERALD, GA., February 10, 1896.

*Editor of Northwestern Lancet:*

SIR:—I am here at the "Old Soldiers' Colony." Have some land which I began plowing to-day to prepare for fruit trees. There are some six or ten thousand Northern people here, having principally come since November, 1895, all busy, happy, healthy. The colony has its 12,000 families, and will take no more members. Minnesota has about four or five hundred here, with several times as many to follow. Minneapolis people are frequently met. The weather is delightful, like late springtime in Minnesota. Air pure and clear, water the finest on earth. No malaria shows yet, and the old Georgian's word and appearance for it, "there is none here." Half the people here came by teams and their long rides from the Dakotas, Kansas, Texas, Ohio, Indiana, Illinois, Iowa, New York, Michigan, and, in fact, from every State in the Union, including many from Idaho and California, have been wearisome.

Very many are here mainly from poor health, and a large percentage of the asthmatics and rheumatoids are greatly improved, and the relief found here from indigestion is most wonderful. This will prove a very poor market for pepsins.

Will the *Lancet* please do me and the Old Soldiers' Colony the justice and great favor to correct, over my signature, a libelous and infamously false statement printed in some of the Minneapolis papers to the effect that "the old soldiers are dying off down there like cattle with the murrain." Although a large proportion of the male adult population are old soldiers, and among them a counterpart of every old cripple and walking invalid pensioner you ever

*The above letter we reproduce, with the permission of the writer, Dr. Coe, from the *Northwestern Lancet*, of St. Paul. It is a full refutation of some of the reports in circulation about the colony. Dr. Coe writes us that "this article was true when I wrote it, and it is true now."

knew, there has not but one old soldier died here from any cause. Of the five deaths which have taken place in the colony two have been from accidental causes, one from marasmus (a bottle-fed infant), and two from lung disease, one an advanced consumptive whose friends hardly expected to reach here with him alive, and another from hemorrhage of the lung, no doubt caused by a hurt in over-lifting in the quarry.

As a sanitary officer of the colony and surgeon in charge of the Colony Hospital, I speak from positive information. The only disease at all common is a continued fever, similar to, though much milder, than our northern typhoid, not inclined to diarrhea or delirium, yet when established generally continues two to four weeks, and if seen in its formative period may quite generally be shortened and frequently averted by the abortive treatment of Woodbridge, of Youngstown, Ohio. These fevers have as their most threatening symptoms the various hemorrhages incident to typhoid. So far as my pessonal observation extends the disease is principally confined to young adults and children, and I do not remember a case following a previous run of typhoid.

The standard of practice is well up in Georgia, and the etiquette of the medical profession is well abreast that of the highest standard, and seems parallel with the polite and chivalric manners justly accorded to our Southern brethren.

S. B. COE, M.D.

